# Explainable and Subject-Independent VO_2_ Estimation Using a Single IMU: A Lightweight Ensemble Framework Under LOSO Validation

**DOI:** 10.3390/s26103062

**Published:** 2026-05-12

**Authors:** Vidyarani K. Rajashekaraiah, Viswanath Talasila, Rashmi Alva, Prem Venkatesan, Ravi Prasad K. Jagannath, Gurusiddappa R. Prashanth

**Affiliations:** 1 Department of Electronics and Communication Engineering, National Institute of Technology, Kottamoll Plateau, South Goa District, Cuncolim 403703, Goa, India; 2 Department of Electronics and Telecommunication Engineering, Center for Imaging Technologies, Ramaiah Institute of Technology, Bengaluru 560054, Karnataka, India; 3 Center for Imaging Technologies, Ramaiah Institute of Technology, Bengaluru 560054, Karnataka, India; 4 Department of Physiotherapy, Manipal College of Health Professions, Manipal Academy of Higher Education, Old Airport Road, Bengaluru 560017, Karnataka, India; 5 Department of Mathematics, National Institute of Technology, Kottamoll Plateau, South Goa District, Cuncolim 403703, Goa, India

**Keywords:** oxygen uptake estimation, heel-strike detection, inertial measurement unit, extreme learning machine, random forest, temporal convolutional network, leave-one-subject-out, SHAP

## Abstract

Continuous estimation of oxygen uptake (VO_2_) using wearable inertial sensors offers a practical alternative to laboratory-based metabolic testing but remains challenging due to the indirect relationship between kinematics and physiological demand. This study presents a lightweight two-stage pipeline for simultaneous heel-strike (HS) detection and VO_2_ estimation using a single calf-mounted IMU. In Stage 1, an Extreme Learning Machine (ELM) + Random Forest (RF) ensemble achieves the highest HS detection F1-score (0.818) under leave-one-subject-out (LOSO) validation, outperforming a temporal convolutional network (TCN) deep learning baseline (F1 = 0.674), which exhibited higher variability across subjects. In Stage 2, kinematic and gait-derived features from 30 s windows are used to estimate normalized VO_2_ via RF and ensemble regression under LOSO cross-validation across 24 participants. The RF model achieves a median R^2^ of 0.687 using predicted HS (Pred-HS) events and 0.679 using ground-truth (GT) annotations, with the ensemble showing similar performance (median R^2^ ≈ 0.675–0.691). No statistically significant difference was observed between GT-HS and Pred-HS conditions (*p* > 0.05). SHAP analysis identifies accelerometer variability (acc_std) and gyroscope-derived features as dominant predictors, with demographic variables contributing minimally. Overall, the results suggest that VO_2_ estimation may be achieved using automatically detected gait events without manual annotation. The proposed pipeline is computationally efficient and indicates feasibility under controlled conditions, subject to further validation.

## 1. Introduction

VO_2_ is the gold-standard indicator of cardiorespiratory fitness and exercise intensity [1]. In clinical settings, VO_2_ is measured with indirect calorimetry using a breath-by-breath metabolic cart, which requires a face mask and dedicated laboratory equipment. Such measurements are impractical for everyday health monitoring, community-based rehabilitation, or large-scale population screening. Wearable IMUs have, therefore, emerged as a promising surrogate, providing continuous accelerometer and gyroscope data at minimal cost [2].

The fundamental challenge lies in bridging the gap between raw IMU kinematics and the physiological construct of VO_2_. Early approaches relied on simple regression models using accelerometer-derived activity counts. More recent work has shifted toward DL approaches for estimating energy expenditure (EE) and VO_2_ from wearable sensor data [3]. Several studies have leveraged machine learning (ML) algorithms—including RF [4], gradient-boosted trees [5], and neural networks [6]—to learn non-linear mappings from features to VO_2_.

A recurring limitation in the literature is the reliance on random data splits, which may overestimate generalization when subject data appear in both training and test sets [7]. Moreover, most existing pipelines depend on manually annotated gait events or subject-dependent protocols, limiting their applicability. Subject-independent evaluation using LOSO cross-validation has been recommended for a more realistic assessment, yet its integration within a fully automated VO_2_ estimation framework has received limited attention.

To address these gaps, this study proposes a two-stage automated pipeline that combines HS detection with VO_2_ estimation using a single IMU, validated under strict LOSO evaluation without manual gait annotation. Statistical analysis confirms no statistically significant difference between GT and Pred-HS conditions (*p* > 0.05), and SHAP-based analysis provides interpretability of the model’s predictions.

This study makes the following key contributions:

(1) An integrated two-stage pipeline combining HS detection and VO_2_ estimation using a single IMU, validated without manual labeling under strict LOSO evaluation.
(2) A statistical analysis demonstrating no statistically significant difference in VO_2_ estimation performance when using automatically detected gait events compared to GT annotations, suggesting that manual labeling may not be required under the evaluated conditions.
(3) A computationally efficient ensemble learning architecture (ELM + RF + XGBoost) demonstrating comparable performance across configurations under LOSO validation.
(4) A comprehensive explainability analysis using SHAP to identify dominant biomechanical predictors of VO_2_ and quantify the limited contribution of demographic variables.

### Literature Review

HS detection from wearable sensors has been extensively studied for gait analysis. Threshold-based methods based on accelerometer magnitude peaks are simple but brittle under speed variations [8]. Supervised classifiers such as Hidden Markov Models [9], RF [10], and Support Vector Machines [11] improve robustness by exploiting multi-axis features. Romijnders et al. [12] demonstrated a convolutional neural network (CNN) for gait-event detection from lower-back IMU data, achieving high accuracy but at considerable computational cost. More recently, transformer-based approaches have further improved sequence modeling for gait analysis, enabling more accurate stride segmentation from IMU signals [13]. This work complements these approaches by proposing a lightweight ensemble that combines the efficiency of an ELM with the stability of tree-based learners.

Survey studies further emphasize the growing role of intelligent wearable systems in health monitoring and sports analytics [14], while learning-based frameworks for activity recognition and movement analysis contribute indirectly to VO_2_ estimation by improving feature representation from wearable signals [15]. Large-scale accelerometer-based studies have also demonstrated the feasibility of quantifying physical activity and EE in free-living conditions, providing a strong foundation for wearable-based physiological modeling [16]. Additionally, recent work has explored IMU optimization and sensor configurations to enhance gait analysis and physiological estimation accuracy [17].

Despite recent advances in IMU-based gait analysis and VO_2_ estimation, several limitations remain. Many existing approaches rely on subject-dependent validation protocols, which may overestimate generalization performance. Additionally, multi-sensor configurations and laboratory-based annotations limit scalability in practical settings. Furthermore, most studies lack model interpretability, making it difficult to understand the relationship between biomechanical signals and physiological responses. Finally, the dependence on manually annotated gait events limits the practicality of wearable systems.

ELM, introduced by Huang et al. [18], is a single-hidden-layer feedforward network in which input-to-hidden weights are randomly assigned and remain fixed. Only the hidden-to-output weights are learned analytically via regularized least squares, resulting in training speeds orders of magnitude faster than backpropagation-based networks. Despite its simplicity, ELM has demonstrated competitive performance in time-series classification [19], biomedical signal processing [20], and human activity recognition [21]. Recent studies have further extended ELM using optimization techniques and hybrid learning frameworks to improve performance in wearable sensing and activity recognition tasks [22]. In this work, ELM serves a dual role: as a base classifier in the HS detection ensemble and as a base regressor in the VO_2_ estimation pipeline.

TCNs apply dilated causal convolutions to capture long-range temporal dependencies without recurrence [23]. Exponentially increasing dilation factors enable a large receptive field with a compact parameter budget. TCNs have been successfully applied to action recognition [24], speech separation [25], and gait-phase estimation [26]. Recent advances in sequence modeling, including TCNs and state-space models, have further improved efficiency in capturing long-range dependencies in time-series data [23,27]. This work addresses this gap by evaluating a two-layer dilated TCN under strict LOSO validation.

SHapley Additive exPlanations (SHAP), proposed by Lundberg and Lee [28], provides theoretically grounded, locally faithful, and globally consistent feature-importance values based on cooperative game theory. TreeSHAP, an efficient variant for tree-based models, computes exact Shapley values in polynomial time [29]. Recent studies have emphasized the importance of explainable AI techniques such as SHAP for interpreting ML models in physiological and biomechanical applications, and it has been widely adopted in biomechanics and clinical decision-support systems to improve model transparency [30,31,32]. In this work, SHAP is used to generate a comprehensive beeswarm visualization of the 20-dimensional feature set to identify dominant predictors of VO_2_.

Table 1 summarizes the key related studies, comparing sensor modality, target variable, model architecture, validation strategy, and reported performance. The table highlights the diversity of approaches and the limited adoption of subject-independent (LOSO) evaluation.

The remainder of this paper is organized as follows. Section 2 presents the materials and methods. Section 3 describes the experimental results. Section 4 discusses the findings and limitations. Section 5 concludes the paper.

## 2. Materials and Methods

### 2.1. Participants and Data Collection

A total of 24 healthy adults (10 male, 14 female; age 21–36 years; height 150–180 cm; weight 44–84 kg) participated in the study as summarized in Table 2. Each participant performed a graded treadmill walking following the Bruce protocol, in which speed and incline are progressively increased at fixed intervals. The protocol comprised five stages, with treadmill speed increasing progressively from 2.7 km/h to 6.0 km/h and incline from 0% to 14%, with each stage lasting approximately three minutes. Participants were instrumented after a standardized five-minute rest period. The test was conducted under supervised laboratory conditions at Manipal Hospital, Bengaluru, with ethical approval obtained from the Ethics Committee of Manipal Hospitals and written informed consent from all participants. Full details of the experimental protocol and data acquisition procedure are described in our prior work [33,34]. A single IMU (tri-axial accelerometer and gyroscope, 100 Hz sampling rate) was attached to the calf muscle of the right leg throughout the test. GT-VO_2_ was measured simultaneously using a breath-by-breath metabolic analyzer. HS events were manually annotated from synchronized force-plate and IMU data.

**Figure 1:**
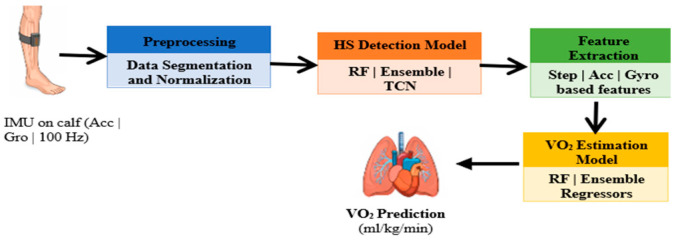
Proposed two-stage IMU-based pipeline for HS detection and VO_2_ estimation.

Figure 1 shows the overview of the proposed two-stage IMU-based pipeline. In Stage 1, HS events are detected using a stacked ensemble model (ELM + RF + XGBoost) under LOSO validation. In Stage 2, 30 s non-overlapping windows are used to extract biomechanical and gait-derived features via an RF regressor. The pipeline operates using only IMU-derived signals during inference, without requiring external reference sensors.

### 2.2. Stage 1—HS Detection

A 15-dimensional feature vector is extracted for each sample using a sliding window of w = 5 samples centered on the current time step. The features comprise the six raw IMU channels (acc_x, acc_y, acc_z, gyro_x, gyro_y, and gyro_z), accelerometer and gyroscope magnitudes, their first-order temporal derivatives, and rolling statistics (mean, standard deviation, max, min, and range of the accelerometer magnitude). The accelerometer magnitude is computed as in (1).

(1) $$\left\|a(t)\right\|=\sqrt{a_{x}{\left(t\right)}^{2}+a_{y}{\left(t\right)}^{2}+a_{z}{\left(t\right)}^{2}}$$

Similarly, the gyroscope magnitude is in (2):

(2) $$\left\|\omega (t)\right\|=\sqrt{\omega_{x}{\left(t\right)}^{2}+\omega_{y}{\left(t\right)}^{2}+\omega_{z}{\left(t\right)}^{2}}$$

The first-order temporal derivatives are approximated by finite differences in (3) and (4).

(3) $$\frac{d\left\|a\right\|}{dt}\approx \frac{\left\|a(t)\right\|-\left\|a(t-1)\right\|}{∆t}$$

(4) $$\frac{d\left\|\omega \right\|}{dt}\approx \frac{\left\|\omega (t)\right\|-\left\|\omega (t-1)\right\|}{∆t}$$

The rolling statistics over the window W are calculated as in (5) and (6).

(5) $$\mu_{a}=\frac{1}{\omega }\sum_{i\epsilon W}\left\|a(i)\right\|$$

(6) $$\sigma_{a}=\sqrt{\frac{1}{\omega }\sum_{i\epsilon W}{\left(\left\|a(i)\right\|-\mu_{a}\right)}^{2}}$$

The concatenated feature vector is shown in (7).

(7) $$x\left(t\right)={\left[\begin{array}{c} a_{x}\left(t\right),a_{y}\left(t\right),a_{z}\left(t\right),\omega_{x}\left(t\right),\omega_{y}\left(t\right),\omega_{z}\left(t\right),\\ \left\|a(t)\right\|,\left\|\omega (t)\right\|,\\ \begin{array}{c} ∆\left\|a\right\|(t),∆\left\|\omega \right\|(t)\\ \mu_{a},\sigma_{a},{max}_{a},{min}_{a},{range}_{a} \end{array} \end{array}\right]}^{T}\in R^{15}$$

#### Three Classifiers Are Employed

- *RF Classifier (RF):* An ensemble of T = 150 decision trees is trained using bootstrap aggregation (bagging). Each tree h_t_ is grown on a bootstrap sample D_t_ with replacement from D. At each internal node, a random subset of m = sqrt(d) features is considered for splitting (where d is the feature dimensionality). The classification decision is obtained by majority voting using (8).
  (8) $${\hat{y}}_{RF}\left(x\right)=mode\left\{h_{t}(x)\right\}\;for\;t=1\ldots \ldots \;T$$
  The out-of-bag (OOB) error provides an unbiased estimate of the generalization error without a separate validation set. Balanced class weights $w_{c}=\;\frac{N}{\left(2\times N_{c}\right)}$ are applied to address label imbalance between HS and non-HS samples.
- *ELM Classifier:* Given N training samples, the ELM randomly assigns input weights $W\in R^{d\times N_{h}}$ and biases ${b\in R}^{N_{h}}$ from a uniform distribution and then computes the hidden-layer output matrix as in (9):
  (9) $$H=g\left(XW+1_{N}b^{T}\right)\;in\;R^{d\times N_{h}}\;$$
  where g(·) = tanh(·) is the activation function and Nh = 150 is the number of hidden neurons. The output weights beta are obtained in closed form via the Moore–Penrose pseudo-inverse as in (10):
  (10) $$\beta =H^{+}y={\left(H^{T}H\right)}^{-1}H^{T}y$$
  For classification, the raw output is mapped to a probability via the sigmoid function as in (11).
  (11) $${\hat{p}}_{ELM}\left(x\right)=\sigma \left(h{\left(x\right)}^{T}\beta \right)=\frac{1}{1+exp(-h{\left(x\right)}^{T}\beta )}\;$$
- *Stacked Ensemble:* The class-probability outputs of RF and ELM are concatenated into a 2-dimensional meta-feature vector, which is then processed by an XGBoost meta-learner to produce the final HS prediction using Equation from (12) to (14).
  (12) $$z=\left[\begin{array}{c} {\hat{p}}_{RF}(x)\\ {\hat{p}}_{ELM}(x) \end{array}\right]\;\in \;R^{2}\;$$
  (13) $${\hat{p}}_{meta}\left(x\right)=\sigma \;\left(\sum_{k=1}^{K}f_{k}(z)\right)$$
  (14) $${\hat{y}}_{HS}=\left\{\begin{array}{c} 1,\;if\;{\hat{p}}_{meta}\left(x\right)\;\geq \tau^{*}\;\\ 0,\;otherwise\; \end{array}\right.$$
  where τ* = 0.5 is the classification threshold. Stacking architecture leverages the complementary strengths of each base learner: ELM contributes fast non-linear projections, RF provides ensemble stability through bagging, and XGBoost captures complex feature interactions via boosting.
- *TCN architecture:* A lightweight temporal convolutional network (TCN) is used as a DL baseline. The model consists of two 1D convolutional layers with kernel size 3 and dilation factors 1 and 2, with increasing channel depth (32 and 64 filters), followed by ReLU activations, adaptive average pooling, and a fully connected output layer. Non-causal dilated convolutions are used to capture short-range temporal dependencies while maintaining computational efficiency. The model is trained using binary cross-entropy loss with logits and optimized using Adam for a fixed number of epochs under LOSO conditions.
  The lightweight architecture prioritizes computational efficiency and is designed to operate effectively under limited training data in LOSO settings.

### 2.3. Stage 2: VO_2_ Estimation

In the second stage, the IMU recording is segmented into non-overlapping 30 s windows (N_w_ = 3000 samples at f_s_ = 100 Hz). For each window, a 20-dimensional feature vector is computed from both kinematic statistics and the HS events detected in Stage 1 (or, alternatively, from GT-HS labels). The target variable is the normalized VO_2_ value from the subsequent 30 s breath, as in (15).

(15) $${VO}_{2,\;norm\;}\left(s\right)=\frac{{VO}_{2}\left(s\right)-\mu }{\sigma_{s}+10^{-6}}$$

where $\mu_{s}$ and $\sigma_{s}$ are the per-subject mean and standard deviation, respectively. Subject-wise normalisation removes inter-individual offset and scale differences, allowing the model to focus on within-subject temporal variation. The normalized VO_2_ represents relative within-subject variation and does not directly correspond to absolute metabolic values. The IMU-derived features are defined mathematically as follows:

Accelerometer magnitude features are shown in Equations (16) to (20).

(16) $$\left\|a\right\|=\sqrt{{a_{x}}^{2}+{a_{y}}^{2}+{a_{z}}^{2}}$$

(17) $$f_{1}={acc}_{mean}=\frac{1}{N_{w}}\sum_{i=1}^{N_{w}}\left\|a_{i}\right\|$$

(18) $$f_{2}={acc}_{std}=\frac{1}{N_{w}}\sum_{i=1}^{N_{w}}{\left(\left\|a_{i}\right\|-{acc}_{mean}\right)}^{2}$$

(19) $$f_{3}={acc}_{rms}=\sqrt{\frac{1}{N_{w}}\sum_{i=1}^{N_{w}}{\left(\left\|a_{i}\right\|\right)}^{2}}\;$$

(20) $$f_{4}={acc}_{range}=\underset{i}{\max}\left\|a_{i}\right\|-\underset{i}{\min}\left\|a_{i}\right\|\;$$

Gyroscope magnitude features are shown in Equations (21) to (23).

(21) $$\left\|\omega_{i}\right\|=\sqrt{{\omega_{x,\;i}}^{2}+{\omega_{y,i}}^{2}+{\omega_{z,i}}^{2}}$$

(22) $$f_{5}={gyro}_{std}=\sqrt{\frac{1}{N_{w}}\sum_{i=1}^{N_{w}}{\left(\left\|\omega_{i}\right\|-\mu_{\omega }\right)}^{2}}$$

(23) $$f_{6}={gyro}_{rms}=\sqrt{\frac{1}{N_{w}}\sum_{i=1}^{N_{w}}{\left(\left\|\omega_{i}\right\|\right)}^{2}}$$

Gait temporal features (derived from HS events) are shown in Equations (24) to (26).

(24) $$f_{7}=N_{HS}=\sum_{i=1}^{N_{w}}HS(i)$$

(25) $$f_{8}=cadence=\frac{N_{HS}}{T_{window}}\times 60\;$$

(26) $$f_{9}=step\_freq=\frac{N_{HS}}{T_{window}}\;$$

Let t_1_, t_2_, …, $t_{N_{HS}}$ be the time indices of detected HS events. The stride intervals are shown in Equations (27) to (29).

(27) $$\delta_{k}=\frac{t_{k+1}-t_{k}}{f_{s}}\;$$

(28) $$f_{10}={stride}_{mean}=\frac{1}{N_{HS}-1}\sum_{i=1}^{N_{HS}-1}\delta_{k}$$

(29) $$f_{11}={stride}_{std}=\sqrt{\frac{1}{N_{HS}-1}\sum_{i=1}^{N_{HS}-1}{\left(\delta_{k}-{stride}_{mean}\right)}^{2}}$$

Jerk features are shown in Equations (30) and (31).

(30) $$jerk(i)\;\approx \;\frac{\left\|a(i)\right\|-\left\|a(i-1)\right\|}{∆t}$$

(31) $$f_{12}={jerk}_{rms}=\sqrt{\frac{1}{N_{w}-1}\sum_{i=1}^{N_{w}}{jerk\left(i\right)}^{2}}$$

Spectral features (computed via Welch PSD with Hanning window, segment length 256) are shown in Equations (32) to (34).

(32) $$S_{xx}\left(f\right)=\frac{1}{K}\;\sum_{k=1}^{K}{\left|F\left\{x_{k}\cdot \;\omega \right\}\;(f)\right|}^{2}$$

(33) $$f_{13}={dom}_{freq}=\arg\underset{f}{\max}S_{xx}$$

(34) $$f_{14}={band}_{power}=\sum_{f\;\in [0.5,\;3.0]}S_{xx}(f)∆f$$

The composite intensity feature is shown in Equation (35).

(35) $$f_{15}=intensity={acc}_{rms}$$

The features are selected based on their biomechanical and physiological relevance. Accelerometer mean and RMS (f_1_, f_3_) reflect average locomotor intensity, which correlates directly with metabolic cost [1,33]. Accelerometer std (f_2_) captures stride-to-stride variability in movement intensity, an indicator of gait irregularity and effort [8,33]. Gyroscope std and RMS (f_5_, f_6_) reflect angular velocity variability at the calf, linked to rotational limb dynamics during walking. Jerk RMS (f_12_) quantifies the rate of change of acceleration, which is associated with the mechanical impulse at heel contact. Spectral features (f_13_, f_14_) capture dominant stepping frequency and rhythmic energy content, both established markers of walking effort and cadence-based VO_2_ demand [1,34].

The complete feature vector is as follows:

(36) $$x=\;{\left[f_{1},\;f_{2}\ldots \ldots \ldots f_{15},\;f_{demo}\right]}^{T}\;\in \;R^{d}$$

where d = 15 for IMU-only and d = 20 when demographic features (age, height, weight, gender,$\;BMI=\;\frac{weight}{{height}^{2}}$) are appended.

#### 2.3.1. RF Regressor

An ensemble of T = 700 bagged decision trees (max depth 18, min samples per leaf 2). Each tree h_t_ is fit on a bootstrap sample D_t_. The final prediction averages all trees as in (37).

(37) $${\hat{y}}_{RF}\left(x\right)=\frac{1}{T}\;\sum_{t=1}^{T}h_{t}(x)$$

The variance of the ensemble prediction decreases with T according to Equation (38).

(38) $$var\left[{\hat{y}}_{RF}\right]=\rho \sigma^{2}+\frac{1-\rho }{T}\;\sigma^{2}\;$$

where ρ is the pairwise correlation between trees and σ^2^ is the individual tree variance. Feature subsampling (m = d/3 at each split) reduces ρ, improving ensemble diversity.

#### 2.3.2. Stacked Ensemble Regressor

Three base regressors produce first-level predictions that are combined by a Ridge meta-learner. The base regressors are as follows:

- RF: as defined above (T = 300, depth = 6).
- XGBoost (M = 300 estimators, depth = 4, ƞ = 0.05): The additive model is $F_{m}\left(x\right)=\;\sum_{m=1}^{M}\eta f_{m}(x)$, where each tree f_m_ is added sequentially as $F_{m}\left(x\right)=\;F_{m-1}\left(x\right)+\;\eta \;f_{m}(x)$. At each iteration, f_m_ is learned by minimizing a second-order Taylor expansion of the squared loss function.
- ELM Regressor (N_h_ = 250, α = 0.2): The hidden layer output of the ELM is given by $H=tanh\left(XW_{rand}+\;1_{N}b_{rand}^{T}\right)$, and the output weights are computed using Tikhonov regularization as in (39).
  (39) $$\beta_{ELM}={\left(H^{T}H+\alpha I\right)}^{-1}H^{T}y$$
  The predictions from the base models are stacked into a meta-feature vector z = [ŷ_RF_, ŷ_XGB_, ŷ_ELM_] $z\in R^{3}$, which is standardized to zero mean and unit variance using training data statistics. A Ridge regression meta-learner is trained by minimizing an L2-regularized squared loss function.

The meta-learner is trained using predictions generated from the training data within each LOSO fold, ensuring that no information from the test subject is used during stacking.

### 2.4. Cadence and Steps per Minute Computation

Cadence (steps per minute) is a clinically important gait parameter that is closely associated with metabolic cost. In this study, cadence is computed from the HS events detected within each 30 s window as in (40).

(40) $$Cadence=\frac{N_{HS}}{T_{window}}\times 60$$

where $N_{HS}$ denotes the number of detected HS events and $T_{window}=30\;s$. Under this setting, cadence simplifies to Cadence $=\frac{N_{HS}}{30}\times 60$.

The step frequency (in Hz) is defined as in (41).

(41) $${Step}_{freq}=\frac{N_{H}}{T_{window}}$$

Stride interval statistics are also extracted, where the mean and standard deviation of inter-HS time intervals within each window quantify gait regularity, which has been shown to correlate with metabolic efficiency [8]. Mechanical intensity is defined as the product of accelerometer root mean square (RMS) and cadence, as in Equation (42), and serves as a composite kinematic proxy for exercise intensity. Across the 24 participants, the mean cadence during treadmill walking was 82.4 steps/min (range: 60–120), consistent with values reported for comfortable walking speeds in the literature.

(42) $$Intensity={acc}_{rms}\times Cadence$$

### 2.5. Leave-One-Subject-Out Validation

LOSO cross-validation is employed to approximate evaluation on unseen individuals, which is relevant for wearable systems where subject-specific calibration may be impractical. In each of the N = 24 folds, data from one subject is reserved for testing, while the remaining 23 subjects are used for training. This protocol prevents any subject-level data leakage and provides a realistic estimate of generalization to new users [7]. For HS detection, performance is evaluated using the F1-score, while VO_2_ estimation is assessed using the coefficient of determination ($R^{2}$) as in Equation (43).

(43) $$R^{2}=1-\frac{\sum_{i=1}^{N}(y_{i}-{\hat{y}}_{i}{)}^{2}}{\sum_{i=1}^{N}(y_{i}-\bar{y}{)}^{2}}$$

where $\bar{y}$ denotes the mean of the GT values in the test set.

To ensure numerical stability, LOSO folds with near-zero target variance ($Var(y_{test})<10^{-4}$) or fewer than three test windows are excluded from aggregate statistics, as such cases can lead to ill-conditioned $R^{2}$ estimates.

### 2.6. GT-HS vs. Pred-HS Experimental Design

A key objective of this study is to determine whether GT-HS labels are required for accurate VO_2_ estimation, or whether the pipeline can operate end-to-end using only Pred-HS events. To investigate this, Stage 2 is executed twice for each model configuration: once using GT-HS and once using HS events predicted by the stacked ensemble in Stage 1 (Pred-HS).

To ensure a fair comparison, the Pred-HS labels ($HS_{pred}$) are stored alongside the GT-HS labels in each subject’s CSV file, and identical windowing is applied in both cases. The $HS_{pred}$ signal is used exclusively for feature extraction in the Pred-HS setting.

### 2.7. Computational Complexity and Deployment Feasibility

The proposed framework is designed with an emphasis on computational efficiency and real-time applicability in wearable systems. The ELM requires only a single matrix inversion during training, avoiding iterative backpropagation and reducing training time.

In this implementation, complete model training requires approximately 8–10 min on a standard workstation, while inference time is less than 5 ms per sample, supporting real-time processing. The final model size is below 0.2 MB, suggesting potential suitability for resource-constrained embedded devices.

Compared to deep learning approaches, which typically involve longer training times and higher memory usage, the proposed ensemble shows comparable performance with lower computational overhead. The use of fixed window-based processing and a low-dimensional feature space (15–20 features) further limits computational requirements.

Although deployment on embedded hardware was not experimentally validated, the low computational complexity and compact model size suggest potential for feasibility under controlled conditions for real-time wearable and edge-device applications. However, this remains to be confirmed through future hardware-level evaluation.

All computational evaluations were performed on a workstation environment. The relatively low computational complexity of ELM and tree-based models suggests potential feasibility for deployment on resource-constrained hardware, although this was not experimentally evaluated.

## 3. Results

### 3.1. Stage 1: HS Detection

**Figure 2:**
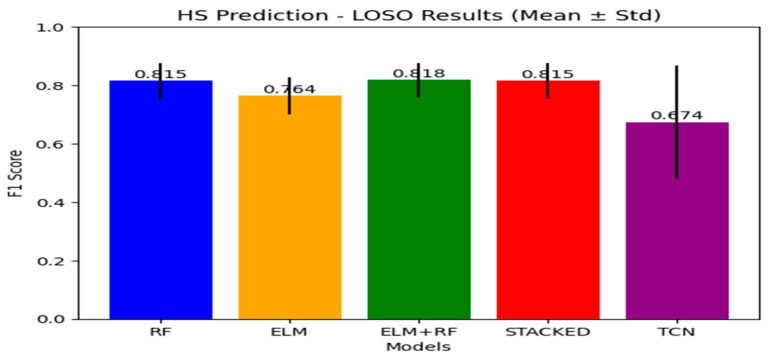
Mean LOSO F1-score comparison for HS detection across RF, ELM, ensemble, stacked ensemble, and TCN.

Table 3 and Figure 2 reports the LOSO HS detection performance across different models. The ELM+RF ensemble achieves the highest mean F1-score (0.818 ± 0.058), followed closely by the RF classifier and stacked ensemble (both 0.815 ± 0.061). However, the improvement over the RF baseline is marginal (~0.3%), suggesting that ensemble methods provide limited performance gains under the LOSO setting. This indicates that the RF model already captures most of the discriminative structure in the data.

The stacked ensemble (ELM+RF+XGB) does not show any improvement over the RF model, achieving identical performance across all subjects. This suggests that the stacking strategy does not effectively leverage complementary information from base learners, possibly due to high correlation between model predictions or suboptimal meta-learning.

The standalone ELM model achieves lower performance (0.764 ± 0.063), indicating weaker generalization compared to tree-based methods. However, its inclusion in the ensemble contributes to model diversity, leading to a slight improvement in the combined model. The TCN model achieves a lower mean F1-score (0.674 ± 0.194) and exhibits substantially higher variability across subjects, with performance ranging from near-perfect detection (0.898) to complete failure (0.000). This instability highlights the sensitivity of DL models to subject-specific variations and limited training data under strict LOSO validation.

Overall, the results indicate that tree-based models (RF and variants) consistently outperform DL approaches in this setting, while ensemble methods provide only marginal and inconsistent improvements.

### 3.2. Stage 2: VO_2_ Estimation with GT-HS

Table 4 presents the VO_2_ estimation performance when GT-HS labels are used for feature extraction under the LOSO protocol. The RF regressor without demographic features achieves a median R^2^ of 0.679, while the ensemble model reaches 0.691. This difference is small and not consistent across configurations. When demographic features are included, the RF model shows minimal change (0.679 → 0.680), whereas the ensemble model shows a slight decrease (0.691 → 0.681).

The mean R^2^ values are lower (≈0.40–0.44), which reflects inter-subject variability and the influence of a small number of low-performing subjects, as the mean is more sensitive to such cases than the median. Overall, both models show similar median performance, with only small differences across feature configurations.

### 3.3. Stage 2: VO_2_ Estimation with Pred-HS

Table 5 presents the VO_2_ estimation results using HS events predicted by the Stage 1 ensemble. The RF regressor achieves a median R^2^ of 0.687, slightly higher than its GT-HS counterpart (0.679), although the difference is small. For the ensemble model, the median R^2^ decreases from 0.691 (GT-HS) to 0.675 (Pred-HS). When demographic features are included, a small reduction in performance is observed for both models under Pred-HS conditions, with RF at 0.622 and the ensemble at 0.626.

The small difference between GT-HS and Pred-HS conditions (ΔR^2^ < 0.02) indicates that the impact of HS source is limited under the evaluated conditions. No statistically significant difference was observed between GT-HS and Pred-HS conditions (*p* > 0.05); however, this does not imply formal equivalence. The lower mean R^2^ reflects the same inter-subject variability discussed in Section 3.2 (see also Section 4.5).

To evaluate the contribution of individual base learners, an ablation study was conducted comparing ELM, RF, XGBoost, and the stacked ensemble under LOSO validation. As shown in Table 6, the stacked ensemble achieves the highest median R^2^ of 0.691 under GT-HS conditions, representing a small increase over RF (0.679) and XGBoost (0.675). Under Pred-HS conditions, RF attains the highest median R^2^ (0.687), while the ensemble shows a slight decrease (0.675).

These results indicate that stacking provides modest improvements in some settings but does not consistently outperform individual models. The performance of ELM is lower (0.466 and 0.281), indicating limited generalization when used alone. Overall, RF and XGBoost show relatively consistent performance, while the ensemble offers small, condition-dependent gains.

### 3.4. Cadence and Steps-per-Minute Analysis

**Figure 3:**
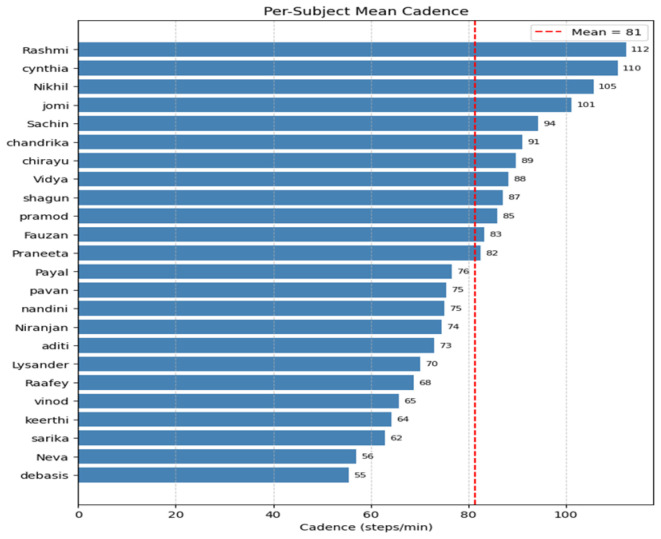
Per-subject mean cadence (steps/min) during treadmill walking, derived from GT-HS events.

Figure 3 illustrates the per-subject mean cadence (steps/min) derived from GT-HS annotations during treadmill walking. The cadence ranges from approximately 55 to 112 steps/min across the 24 participants, with a cohort mean of approximately 81 steps/min. This range corresponds to comfortable-to-brisk treadmill walking speeds. The inter-subject variability in cadence reflects differences in leg length, preferred walking speed, and gait biomechanics.

**Figure 4:**
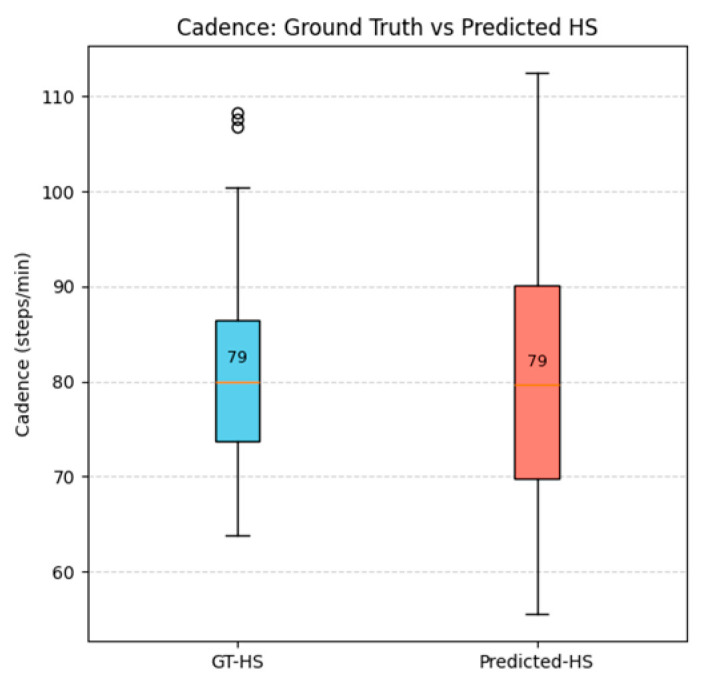
Box-plot comparison of cadence from GT-HS vs. Pred-HS events.

Figure 4 presents a box-plot comparison of cadence computed from GT-HS versus Pred-HS events. The Pred-HS events yield a cadence distribution that closely matches GT annotations, with identical median values (~79 steps/min) and substantial overlap between the two conditions. A slightly wider spread is observed in the Pred-HS distribution, indicating minor variability in event detection. However, the central tendency remains consistent, confirming that the Stage 1 model preserves the temporal gait structure with high fidelity.

### 3.5. GT-HS vs. Pred-HS Comparison

**Figure 5:**
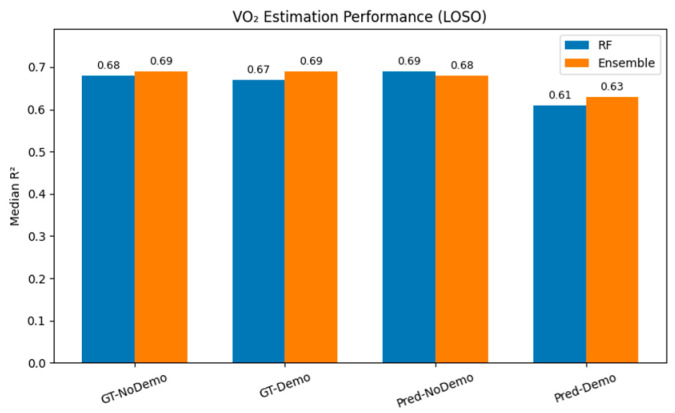
Grouped bar chart comparing median LOSO R^2^ for GT-HS vs. Pred-HS across all model configurations.

Table 7 directly compares GT-HS and Pred-HS results for the RF and ensemble regressors, with and without demographic features; results are also visualized in Figure 5.

### 3.6. SHAP Feature Importance Analysis

**Figure 6:**
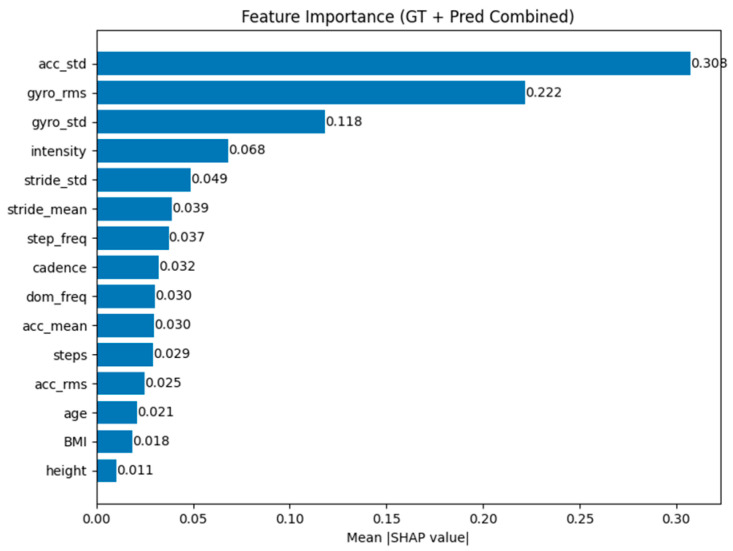
Feature importance for VO_2_ prediction (RF regressor) ranked by mean |SHAP| value.

Figure 6 presents the SHAP-based feature importance analysis. The results identify accelerometer variability (acc_std) and gyroscope-derived features as dominant predictors. The importance of acc_std can be attributed to its ability to capture stride-to-stride fluctuations in locomotor intensity, which are closely related to changes in mechanical effort and metabolic demand during walking; higher variability often reflects increased energetic cost or less stable gait patterns. Gyroscope-derived features capture angular velocity dynamics of the calf segment, encoding rotational motion during stance and swing phases, which directly reflects lower-limb movement mechanics and exercise intensity. Intensity- and stride-related features rank next, consistent with their role as kinematic proxies for overall activity level and gait rhythm. These features are physiologically meaningful, as increased acceleration variability and rotational motion are associated with higher muscular effort and EE. In contrast, demographic variables contribute minimally, suggesting that IMU-derived signals already capture most of the inter-subject variation relevant to VO_2_ estimation in this setting.

### 3.7. Statistical Analysis

To evaluate whether the observed differences across configurations are statistically significant, paired comparisons were performed using both the paired *t*-test and the Wilcoxon signed-rank test across LOSO folds shown in Table 8. Effect sizes were quantified using Cohen’s d.

Paired statistical tests were conducted to assess the significance of performance differences across configurations. No statistically significant difference was observed between GT-HS and Pred-HS conditions (*p* > 0.05) for both the RF and ensemble models (RF: *p* = 0.65, Ensemble: *p* = 0.54), with small effect sizes (|d| < 0.15).

Similarly, the inclusion of demographic features does not show consistent improvements, with all comparisons yielding non-significant results (*p* > 0.05). The closest to significance is observed for the ensemble model with predicted features (Wilcoxon *p* ≈ 0.07); however, this does not meet the significance threshold and is associated with a negligible effect size, indicating no practically meaningful difference.

Normality of paired differences was assessed using the Shapiro–Wilk test. Both parametric (paired *t*-test) and non-parametric (Wilcoxon signed-rank test) analyses are reported. These findings are consistent with the observed similarity between GT-HS and Pred-HS conditions under the evaluated setting.

It is important to note that non-significance does not imply equivalence between conditions; formal equivalence testing (e.g., the Two One-Sided Tests procedure, TOST) was not conducted, and the observed differences should, therefore, be interpreted with appropriate caution.

## 4. Discussion

The strong performance of RF compared to the stacked regressor suggests that ensemble diversity must be balanced against stability in low-data regimes. While stacking introduces model heterogeneity, it may also amplify variance when base learners (e.g., ELM) exhibit stochastic behavior. This highlights the importance of bias–variance trade-offs in subject-independent wearable modeling.

### 4.1. Impact of Pred-HS Events on VO_2_ Estimation

The results indicate that Pred-HS events achieve performance comparable to GT annotations, rather than consistently outperforming them. Although the RF model shows a slight improvement under the no-demographic setting (+0.008), this trend is not consistent across models or configurations. In particular, the ensemble model and demographic feature configurations show minor and inconsistent changes in performance when using Pred-HS.

Statistical analysis indicates no statistically significant difference (*p* > 0.05) between GT-HS and Pred-HS conditions, suggesting that Pred-HS events may retain sufficient temporal information for VO_2_ estimation under the evaluated conditions. Improvements observed in some lower-performing subjects may reflect reduced sensitivity to annotation variability in certain cases. This may be partly explained by the use of 30 s window-based feature aggregation, where VO_2_ estimation relies on summary statistics such as mean, variance, and cadence rather than precise event timing, which may reduce sensitivity to small temporal misalignments in HS detection.

### 4.2. Comparison of TCN and Ensemble Models for HS Detection

The TCN indicates lower and more variable LOSO performance (mean F1 = 0.6741 ± 0.1938) compared to the RF and ensemble models (≈0.815–0.818). Although the performance gap is smaller than initially observed, the TCN exhibits substantially higher variability across subjects, with performance ranging from near-zero to high accuracy. This indicates sensitivity to subject-specific gait patterns under strict LOSO validation. The observed performance difference can be attributed to the nature of feature representation and data availability. The feature-based models (RF and ensemble) rely on handcrafted biomechanical descriptors such as rolling statistics, magnitude derivatives, and axis-wise signals, which explicitly encode gait characteristics relevant to HS events. These representations provide strong inductive bias, enabling better generalization across subjects in small-data settings.

In contrast, the TCN operates directly on raw IMU sequences and learns these representations implicitly. Under the LOSO protocol, the limited training data available in each fold may constrain its ability to learn subject-independent temporal patterns, which can lead to variability in performance across individuals.

It is important to note that this result should not be interpreted as a limitation of DL models in general. Rather, it reflects the constraints imposed by strict subject-independent evaluation and limited dataset size. DL models typically require larger and more diverse datasets to achieve stable generalization. Under such conditions, lightweight feature-based approaches remain more reliable and computationally efficient for wearable applications. The TCN configuration used here is intentionally lightweight and may not reflect the full potential of deep learning architectures when trained on larger and more diverse datasets.

### 4.3. Influence of Demographic Features

Across all configurations, the inclusion of demographic features results in minimal or inconsistent changes in performance. While marginal and inconsistent changes are observed under GT-HS conditions, a modest reduction in performance is observed under Pred-HS settings for both RF and ensemble models.

This suggests that IMU-derived kinematic features effectively capture subject-specific physiological characteristics, reducing the need for additional demographic inputs. This conclusion is further supported by the feature importance analysis, where demographic features contribute minimally compared to gait-derived features.

Although the dataset consists of 24 participants, the use of LOSO cross-validation ensures strict subject-independent evaluation, which is more challenging and realistic than random data splits. Similar dataset sizes have been reported in prior IMU-based physiological estimation studies involving controlled experimental protocols. The consistent median performance across folds suggests stable generalization under LOSO validation despite limited sample size.

### 4.4. Limitations and Future Work

Several limitations should be noted. First, the dataset is limited to 24 participants comprising young, healthy adults performing treadmill walking, which may restrict generalizability to elderly or clinical populations, overground gait, and real-world conditions. Second, the VO_2_ signal is normalized per subject; therefore, the model primarily captures relative temporal variations rather than absolute metabolic values. Third, the TCN configuration is intentionally lightweight to maintain computational comparability; larger architectures with extended training may improve performance but would likely increase computational cost and reduce feasibility under controlled conditions.

Additionally, emerging architectures such as Transformer-based models and state-space sequence models may provide improved representation learning for time-series data. Finally, validation in free-living environments is required to assess real-world applicability and clinical relevance.

### 4.5. Inter-Subject Variability

A key observation in this study is the presence of inter-subject variability. While many subjects achieve relatively high performance (R^2^ > 0.6), a small subset exhibits low or negative R^2^ values. These cases reduce the mean R^2^ more noticeably while having less effect on the median, contributing to the difference between median (~0.68–0.69) and mean (~0.40–0.45) performance observed across experiments. Median R^2^ is preferred in LOSO settings because it is less sensitive to outliers and provides a more reliable estimate of typical subject-level performance.

Potential contributors to lower subject-level performance include individual gait variations such as asymmetric or irregular HS patterns, possible inconsistencies in sensor placement, and inter-individual differences in physiological VO_2_ response under similar mechanical loading. Future work with larger and more diverse cohorts may help further examine these factors.

## 5. Conclusions

This study presents a two-stage framework for HS detection and VO_2_ estimation using a single calf-mounted IMU under a leave-one-subject-out protocol. The ELM+RF ensemble shows marginally improved HS detection performance compared to RF, while the stacked model achieves comparable results, suggesting that feature-based approaches can be effective in limited-data settings.

For VO_2_ estimation, both RF and ensemble models show similar performance, with median R^2^ values in the range of 0.67–0.69. Importantly, no statistically significant difference was observed between GT-HS and Pred-HS conditions (*p* > 0.05). These findings do not imply formal equivalence but indicate that VO_2_ estimation can be performed using automatically detected gait events under the evaluated conditions. Demographic features provide limited additional benefit, suggesting that gait-derived kinematic features already capture much of the subject-specific information. While most subjects achieve relatively high performance, inter-subject variability is observed, highlighting the importance of median-based evaluation in LOSO settings.

Overall, the proposed framework is computationally efficient and indicates potential for wearable physiological monitoring under controlled conditions, subject to further validation.

## Figures and Tables

**Table 1:** Comparative summary of recent and representative studies in IMU-based gait analysis and wearable VO_2_ estimation.

Study	Sensor	Target	Model	Validation	Best Metric
Jung et al. [3] (2025)	Wearable IMU	EE/VO_2_	Deep Learning	Subject-independent	R^2^ ≈ 0.70
Yang et al. [14] (2024)	Multi-sensor (survey)	Physiological monitoring	Survey (ML overview)	—	—
Romijnders et al. [12] (2022)	Back IMU	Gait event	CNN	LOSO	F1 = 0.95
Swathi et al. [15] (2025)	Camera/IMU	Human activity analysis/fitness monitoring	DL (pose-based)	Experimental	—
Ashraf et al. [26] (2024)	Shank IMU	Gait event	TCN	Experimental	F1 ≈ 0.90
Troiano et al. [16] (2014)	Waist Acc	Physical activity/EE	Statistical	Large-scale study	—
Zhao et al. [13] (2025)	Foot IMU	Stride segmentation	Transformer	LOSO	F1 = 0.97
Wilkinson et al. [32] (2026)	—	Explainable AI	XAI Survey	—	—
Yuan et al. [17] (2026)	IMU (running)	Gait analysis	ML (sensor fusion)	Experimental	—
This study	Calf IMU	HS + VO_2_	ELM + RF + XGB	LOSO	R^2^ ≈ 0.68

**Table 2:** Participant characteristics.

Parameter	Value
Subjects	24 (10 M/14 F)
Age (years)	21–36 (mean 24.6)
Height (cm)	150–180 (mean 165.1)
Weight (kg)	44–84 (mean 62.3)
IMU Sampling Rate	100 Hz
IMU Placement	Calf muscle (right leg)
VO_2_ Measurement	Breath-by-breath metabolic analyzer

**Table 3:** LOSO HS detection performance (F1-score).

Model	Mean LOSO F1	Std Dev	Best Subject F1	Worst Subject F1
RF Classifier	0.815	0.061	0.929	0.677
ELM	0.764	0.063	0.934	0.638
ELM + RF (Ensemble)	0.818	0.058	0.934	0.683
Stacked Ensemble (ELM+RF+XGB)	0.815	0.061	0.893	0.677
TCN (2-layer)	0.674	0.194	0.898	0.000

**Table 4:** VO_2_ estimation with GT-HS (LOSO).

Condition	Median R^2^	Mean R^2^
IMU Only + RF	0.679	0.434
IMU Only + Ensemble	0.691	0.409
IMU + Demo + RF	0.680	0.443
IMU + Demo + Ensemble	0.681	0.413

**Table 5:** VO_2_ estimation with Pred-HS (LOSO).

Condition	Median R^2^	Mean R^2^
RF (No Demo)	0.687	0.446
Ensemble (No Demo)	0.675	0.429
RF + Demo	0.622	0.444
Ensemble + Demo	0.626	0.440

**Table 6:** Ablation study for VO_2_ estimation (median LOSO R^2^, no demographics).

Model	GT-HS	Pred-HS
ELM	0.466	0.281
XGBoost	0.675	0.672
RF	0.679	0.687
Stacked Ensemble	0.691	0.675

**Table 7:** GT-HS vs. Pred-HS comparison for VO_2_ estimation (median LOSO R^2^).

Model	HS Source	Median R^2^	ΔR^2^ (Pred − GT)
RF (No Demo)	GT-HS	0.679	+0.008
	Pred-HS	0.687	
Ensemble (No Demo)	GT-HS	0.691	−0.016
	Pred-HS	0.675	
RF + Demo	GT-HS	0.680	−0.058
	Pred-HS	0.622	
Stacked Ensemble + Demo	GT-HS	0.681	−0.055
	Pred-HS	0.626	

**Table 8:** Statistical comparison across configurations.

Comparison	Median A	Median B	*t*-Test (p)	Wilcoxon (p)	Cohen’s d
RF: GT vs. Pred	0.679	0.687	0.651	0.643	−0.098
ENS: GT vs. Pred	0.691	0.675	0.544	0.520	−0.131
RF Demo (GT)	0.679	0.680	0.540	0.329	−0.133
RF Demo (Pred)	0.687	0.622	0.774	0.160	0.062
ENS Demo (GT)	0.691	0.681	0.765	0.300	−0.065
ENS Demo (Pred)	0.675	0.626	0.733	0.070	−0.074

## Data Availability

The datasets generated and/or analyzed during the current study are available from the corresponding author upon reasonable request.
